# Gurka *vs* Slaughter equations to estimate the fat percentage in children with cerebral palsy from all subtypes and levels of the Gross Motor Function Classification System

**DOI:** 10.1186/s12887-023-03970-x

**Published:** 2023-04-01

**Authors:** Jorge A. García-Íñiguez, Andrea A. García-Contreras, Edgar M. Vásquez-Garibay, Alfredo Larrosa-Haro

**Affiliations:** 1 Instituto de Nutrición Humana, Centro Universitario de Ciencias de la Salud, Universidad de Guadalajara, Guadalajara, Jalisco México 44340; 2 Escuela de Nutrición, Universidad Autónoma de Guadalajara, Guadalajara, Jalisco México 45129

**Keywords:** Cerebral palsy, Children, Fat percentage, Slaughter equation, Gurka equation, Bioelectrical impedance analysis

## Abstract

**Background:**

Body composition assessment in children with cerebral palsy (CP) is a challenge, specially the fat percentage. There are different methods that can be used to estimate the fat percentage in this population, such as anthropometric equations, but there is still a need to determine which is the best and most accurate. The purpose of the study was to determine the method that best estimates the fat percentage in children from all CP subtypes and levels of the Gross Motor Function Classification System (GMFCS).

**Methods:**

Analytical cross-sectional study in which 108 children with CP diagnosed by a pediatric neurologist were included with any type of dysfunction and from all levels of the GFMCS. Slaughter equation, Gurka equation and Bioelectrical impedance analysis (BIA) as reference method, were used. Groups were stratified by sex, CP subtypes, GMFCS level and Tanner stage. Median differences, Kruskal–Wallis, Mann–Whitney U test, Spearman's correlation coefficients and simple regressions were used, also multivariate models were performed.

**Results:**

The Slaughter equation differed from the other methods in the total population and when it was compared by sex, CP subtypes, gross motor function and Tanner stage. The Gurka equation showed significant differences by sex and gross motor function. Gurka equation correlated positively and significantly with BIA to estimate the fat percentage in all the CP subtypes and levels of the GMFCS. Tricipital skinfold (TSF), arm fat area (AFA) and weight for age index (W/A) showed the highest variability with respect to fat percentage.

**Conclusion:**

Gurka equation is more appropriate and accurate than Slaughter equation to estimate the fat percentage in children with CP from all subtypes and levels of the GMFCS.

## Background

The assessment of nutritional status in children with cerebral palsy (CP) is not simple [1]. Therefore, the measurement and evaluation of body composition should be considered an essential and routine indicator in the evaluation, assessment, and nutritional intervention of these children [2–6]. Children with CP, especially those with a more serious condition, such as spastic quadriplegia, often have protein-energy malnutrition [7], which can lead to changes in body composition; for example, decreased lean mass (LM), fat mass (FM), and fat percentage, increase in total body water (TBW), and changes in bone mineral density (BMD) [8–10]. There are different methods of measuring and estimating body composition in children with CP; however, there is no consensus that specifies which method is the best of them [11, 12]. Fat percentage is the most clinically applicable measurement for assessing nutritional status [13]. In boys, a low fat percentage is considered to be ≤ 10%, normal is from 11 to 25% and high is > 25%, while in girls, ≤ 15% is considered low, normal is from 16 to 30% and high is > 30% [14]. Dual X-ray absorptiometry (DXA) is a reliable method for accurately estimating FM, LM, fat percentage and bone mass (BM) in children [15–17] and adults [18]; however, it is expensive, requires specialized equipment, and emits low levels of radiation [8, 19–21]. Doubly labeled water (DLW) has been used to assess fat mass in children with CP, and similar to DXA, it is a method that requires specialized equipment, is time-consuming to perform and is commonly used for investigation purposes and in research centers [8, 21]. Two methods that are less expensive and relatively easy to perform, and that can provide valid and reliable measurements for body composition assessment and fat percentage specifically are bioelectrical impedance analysis (BIA) and anthropometry (with two and four skinfolds) [9, 19]. BIA is a fast, noninvasive, portable method that is increasingly used in clinical settings and in studies in children [4, 11, 19, 20]. From a clinical and practical point of view, anthropometry is the most widely used method for estimating body composition since it requires only a skinfold caliper, previously standardized personnel, and the cooperation of the patient [9, 22]. However, there is concern that these methods may not be reliable in children with CP. On the one hand, the hydroelectrolytic status of these children can influence the results obtained by BIA [19]. On the other hand, the known alterations in body composition in children with CP, such as the increase in total body water and the decrease in LM, FM and BMD, indicate that the Slaughter equation underestimates the fat percentage [8]. Although this equation is commonly used in clinical and research settings to estimate body fat percentage in healthy populations, it has been analyzed in children with neurological damage [23]. Gurka et al. [8] published a correction factor that improves the validity of the Slaughter's equations, and some authors recommend the use of the correction factor for children with CP [21]. ESPGHAN guidelines highlight the importance of the routine use of skinfold thickness measurements with the calculation of fat percentages using this equation [12]. Therefore, the purpose of this study is to determine the anthropometric method that best estimates the fat percentage in children with CP from all subtypes and levels of the Gross Motor Function Classification System (GMFCS).

### Materials and methods

In an analytical cross-sectional design study, 108 participants (53 girls and 55 boys) aged 24 months to 16 years 9 months (7 y 9 m ± 4 y 3 m) were included. The participants had CP with any subtypes, and their gross motor function scale level was diagnosed and classified by a pediatric neurologist who attended the pediatric outpatient clinic of the Hospital Civil de Guadalajara “Dr. Juan I. Menchaca”. The participants were fed orally or by gastrostomy feeding tubes according to oral tolerance and severity of malnutrition. The sample size was obtained from a previous study on the nutritional status of children with CP and estimated with a confidence level of 95% (α = 0.05) [24]. Patients with diagnoses unrelated to CP (Down syndrome, autism, degenerative disorders), those receiving medications that could alter body composition (steroids, thyroxine, antiretroviral drugs) and/or those with CP of postnatal origin (traumatic injuries, accidents, tumors, other injuries) were excluded.

### Anthropometry

All measurements were obtained by two previously standardized observers. Weight was obtained with a clean diaper and as little clothing as possible; and a SECA scale (Model 700 Hamburg Germany) with a precision of 50 g was used. The child was weighed in the arms of a family member or an observer, and then only the adult was weighed. The result was obtained by calculating the difference between the weight obtained with the family member and the child, minus the weight of the family member alone. Height (cm) was obtained using leg length (LL) as an alternative height measurement with the following equation: [(3.26 × LL) + 30.8]. The measurement of leg length was conducted using a tape measure (Seca, 206, Hamburg, Germany), according to the technique of Stevenson [25] (1995), and the final value was obtained by averaging the two measurements. Mid-upper arm circumference (MUAC) was measured with a flexible tape (Seca, 206, Hamburg, Germany) at the midpoint of arm length from acromion to olecranon in the left arm. The tricipital skinfold (TSF) was measured with a Lange caliper (Cambridge, Maryland) on the postero-medial part of the left arm at the same point as the MUAC. With the separation of the skin and adipose tissue and the placement of the forceps in the fold one centimeter above the marked point, three measurements were taken per observer and the average was obtained. The subscapular skinfold (SSF) was located at the lower angle of the left scapula where a mark was made; the skin and adipose tissue were taken one centimeter below and diagonally to the mark; the tips of the caliper were placed and after taking the measurement three times, the average was obtained.

The fat percentage was estimated by means of the equations of Slaughter (1988) [26]; when the sum of TSF and SSF was < 35 mm:

- Boys: %fat = 1.21 (∑ two folds) – 0.008 (∑ two folds)2–1.7
- Girls: %fat = 1.33 (∑ two folds) – 0.013 (∑ two folds)2–2.5

If the sum of TSF and SSF was > 35 mm:

- Boys: %fat = 0.783 (∑ two folds) + 1.6
- Girls: %fat = 0.546 (∑ two folds) + 9.7

The correction factor proposed by Gurka et al. (2010) [8] (Table 1) for the Slaughter equation [26] (1988) was calculated. Pubertal stage was determined by the Tanner classification (Tanner, 1962) [27] by a pediatrician with the mother's permission. Stages I and II were considered prepubertal, stage III pubertal, and stage IV and V postpubertal according to the Gurka´s classification (Gurka, 2010) [8].

**Table 1:** Correction factors according to Gurka et al. (2010) [8]

General correction	+ 12.2
Additional correction for:	
Males	-5.0
More severe GMFCS	+ 5.1
Black	-3.1
Pubescent (Tanner stage III)	+ 2.0
Postpubescent (Tanner stages IV and V)	-4.6
Sum skin folds (triceps, subscapular) > 35 mm	-3.2

*GMFCS* Gross Motor Function Classification System. To use these corrections: Add 12.2 to the estimated body fat percentage. If the child with CP is classified within one of the categories, add the respective correction. For example, for an adolescent at GMFCS level 1 whose tricipital and subscapular folds add up to < 35 mm: estimated body fat percentage + 12.2—5.0 + 2.0

### Bioelectric impedance analysis (BIA)

Bioelectrical impedance was performed with Quadscan 4000 equipment (Body Stat Limited, England). After a previous fast of three hours, two electrodes (pediatric) were placed on the back of the hand (at the level of the wrist and metacarpus) and two on the back of the foot (metatarsal and ankle). Metallic objects that could interfere with the measurement were removed, and the procedure was performed at 50 Ohms. The measurement was obtained with the child in the dorsal supine position and as relaxed as possible. The estimation of fat percentage was obtained.

### Ethical considerations

The study did not put the study subjects at risk, and it adhered to the guidelines of the Declaration of Helsinki and the principles of beneficence, nonmaleficence, justice, and autonomy of decision. The Ethics Committee of the Hospital Civil de Guadalajara “Dr. Juan I. Menchaca” approved the protocol under number No. 1344/14.

### Statistical analysis

For descriptive statistics, medians and standard deviations were used for quantitative variables, and frequencies and percentages were used for qualitative variables. For analytical statistics, median differences, Kruskal–Wallis and Mann–Whitney U tests were performed. Spearman's rank correlation coefficients was used. Simple regressions was used to predict anthropometric variables in which a significant correlation was found with the different methods to estimate the fat percentage. Multivariate models were performed. Statistical analysis was performed using SPSS software version 21, and a p value < 0.05 was considered significant.

## Results

Table 2 shows the general characteristics of the participants. It is shown that the percentage of fat estimated with the Gurka equation and with BIA were similar, while this percentage was lower with the Slaughter equation. There was a significant difference when comparing the fat percentages of the total population with the three methods (*p* < 0.001). When comparing the fat percentage in all subjects between the Slaughter and Gurka equations and between the Slaughter equation and BIA, there was a significant difference (*p* < 0.001), while the fat percentage was similar between the Gurka equation and BIA. When comparing the fat percentage by sex, there was a significant difference with the Gurka equation (*p* < 0.001).

**Table 2:** Characteristics of the population

Quantitative variables	All (*n* = 108)	Boys (*n* = 55)	Girls (*n* = 53)	Boys vs. girls p*
Age (months)	80.5 (81.8)	83 (86.0)	80 (87)	0.763
Weight (kg)	15 (9.0)	15.3 (8.6)	13.3 (9.1)	0.178
Height (cm)	103 (26.6)	107 (24.2)	101 (26.9)	0.288
BMI (Z)	-2.3 (3.3)	-1.98 (3.16)	-2.6 (3.3)	0.832
Fat percentage (Slaughter equation)	9.6 (7.3)	9.6 (6.3)	10.0 (7.8)	0.756
Fat percentage (Gurka equation)	23.8 (8.6)	21.9 (6.3)	25.7 (8.3)	< 0.001
Fat percentage (BIA)	27.6 (21.4)	25.8 (22.7)	26.7 (21.7)	0.726

Data are presented as median (interquartile range = percentile 75—25). ^*^Mann–Whitney U test. All: BIA vs. Gurka *p* > 0.05; BIA vs. Salughter *p* < 0.001; Slaughter vs. Gurka *p* < 0.001

Most of the subjects presented spastic CP (73%) and were grouped into levels 4 and 5 according to the GMFCS. Regarding pubertal development, 72% belonged to levels I and II on the Tanner scale (Table 3).

**Table 3:** Qualitative variables of the population

Variable	*n* (%)
Sex	
Female	53 (49)
Male	55 (51)
Pubertal stage	
I and II	78 (72)
III	18 (17)
IV and V	12 (11)
CP subtype	
Spastic	79 (73)
Ataxic	1 (0.9)
Dyskinetic	3 (2.8)
Hypotonic	11 (10.2)
Mixed	14 (13)
GMFCS	
1	5 (4.6)
2	6 (5.6)
3	2 (1.9)
4	16 (14.8)
5	79 (73.1)

Table 4 shows the fat percentage estimated with anthropometry (Slaughter equation and Gurka equation) [8, 26] and BIA in the total population with spastic CP and with other types of dysfunction. Slaughter equation underestimated the fat percentage in both groups when it was compared to BIA, while Gurka equation was closer to BIA.

**Table 4:** Fat percentage according to the type of dysfunction

Type of disfunction	Fat percentage by Slaughter equation	Fat percentage by Gurka equation	Fat percentage by BIA	p*	Median difference (Slaughter—BIA)	Median difference (Gurka—BIA)
Spastic (*n* = 79)	10.0 (7.5)	24.0 (7.5)	28.6 (19.4)	< 0.001	-18.6	-4.6
Other** (*n* = 29)	9.6 (9.1)	23.5 (11.3)	19.7 (23.7)	< 0.001	-10.1	3.8
Subclassification:						
Ataxic (*n* = 1)	6.2 (0.0)	23.5 (0.0)	21.6 (0.0)	-	-15.4	1.9
Dyskinetic (*n* = 3)	9.5 (1.5)	26.8 (8.6)	18.9 (14.6)	0.664	-9.4	7.9
Hypotonic (*n* = 11)	11.7 (8.5)	27.0 (9.7)	28.5 (24.8)	0.012	-16.8	-1.5
Mixed (*n* = 14)	6.1 (8.6)	20.5 (11.9)	15.4 (33.4)	< 0.001	-9.3	5.1

Data are presented as median (interquartile range = percentile 75—25). *Kruskal–Wallis; **Ataxic, dyskinetic, hypotonic and mixed. Mann–Whitney U test (spastic group): Slaughter vs. Gurka *p* < 0.001; Slaughter vs. BIA *p* < 0.001; Gurka vs. BIA *p* = 0.175; "other" group: Slaughter vs. Gurka *p* < 0.001; Slaughter vs. BIA *p* < 0.001; Gurka vs. BIA *p* = 0.267

The analysis of the fat percentage with each method and the GMFCS levels are presented in Table 5. There was a significant difference when comparing the fat percentages obtained by the three methods in levels 1–3 and levels 4–5. When comparing the percentage of fat between levels 1–3 vs. 4–5, the differences were 0.4% with the Slaughter equation (in favor of levels 1–3), 4.9% with the Gurka equation (significant difference) and 1.4% with BIA (in the latter two in favor of levels 4–5). Slaughter equation underestimated the fat percentage in both groups when it was compared to BIA, while Gurka equation was closer to BIA.

**Table 5:** Fat percentage according to the GMFCS

	Fat percentage by Slaughter equation	Fat percentage by Gurka equation	Fat percentage by BIA	p*	Median difference (Slaughter – BIA)	Median difference (Gurka – BIA)
Levels 1–3 (*n* = 13)	10.0 (6.6)	19.7 (6.5)	21.6 (18.6)	< 0.001	-11.6	-1.9
Sublcassification:						
Level 1 (*n* = 5)	11.7 (4.8)	20.9 (6.3)	32.3 (26.9)	0.059	-20.6	-11.4
Level 2 (*n* = 6)	5.6 (7.3)	16.6 (7.5)	19.5 (10.4)	0.011	-13.9	-2.9
Level 3 (*n* = 2)	10.6 (3.1)	22.9 (3.2)	39.3 (6.5)	0.102	-28.7	-16.4
Levels 4–5 (*n* = 95)	9.6 (7.5)	24.6 (9.1)	28.4 (21.5)	< 0.001	-18.8	-3.8
Subclassification:						
Level 4 (*n* = 16)	10.6 (6.6)	26.0 (8.7)	28.9 (24.2)	< 0.001	-18.3	-2.9
Level 5 (*n* = 79)	9.6 (7.5)	24.3 (8.1)	27.5 (21.5)	< 0.001	-17.9	-3.2

Data are presented as median (interquartile range = percentile 75—25). *Kruskal–Wallis; Mann Whitney U test levels 1–3: Slaughter vs. Gurka *p* < 0.001; Slaughter vs. BIA *p* < 0.001; Gurka vs. BIA *p* = 0.208; levels 4–5: Slaughter vs. Gurka *p* < 0.001; Slaughter vs. BIA < 0.001; Gurka vs. BIA *p* = 0.661. **Mann–Whitney U test. Levels 1–3 vs 4–5: Slaughter *p* = 0.459; Gurka *p* < 0.001; BIA *p* = 0.746

Table 6 shows the correlation coefficients between Gurka vs. BIA and Slaughter vs. BIA. It is shown that the fat percentage estimated by Gurka correlated positively and moderately with BIA in all the CP subtypes and in all levels of the GMFCS. Slaugther equation correlated positively with BIA only in the spastic group and in the GMFCS levels 4–5.

**Table 6:** Correlation coefficients between Gurka vs. BIA and Slaughter vs. BIA

	Gurka vs. BIA			Slaughter vs. BIA		
	*r*^a^	*R*^2^	*p*	*r*^a^	*R*^2^	*p*
CP subtypes						
Spastic	0.387	0.150	< 0.001	0.507	0.257	< 0.001
Others^b^	0.572	0.327	0.001	0.361	0.130	0.055
GMFCS						
1–3	0.632	0.399	0.020	0.462	0.213	0.112
4–5	0.403	0.162	< 0.001	0.455	0.207	< 0.001

^a^Spearman´s rank correlation coefficients ^b^Others: ataxic, dyskinetic, hypotonic, and mixed

When comparing the fat percentage among the three groups by Tanner stages, both the Gurka equation and BIA indicate that the group with the lowest fat percentage is that of stages IV-V. When performing post hoc tests to compare the fat percentage between the Tanner stages for each method with the total population, there was a significant difference between stages I-II vs. IV-V with the Gurka equation (*p* = 0.038), between stage III vs. IV-V with the Gurka equation (*p* = 0.038), between stages I-II vs. III with BIA (*p* = 0.016) and between stages I-II vs. IV-V with BIA (*p* = 0.007). When comparing the fat percentage between boys and girls, it was significantly higher in girls in stages I-II (*p* = 0.001) and in stage III (*p* = 0.003) according to the Gurka equation (Table 7). Consistent with the criteria of Lohman et al. (1987) [28] with the Slaughter equation, girls and boys had a low fat percentage both in the total population and when separating the population by Tanner stages. While with the Gurka equation, the fat percentage was found to be adequate in boys and girls. With BIA, it was found to be high in boys in stages I and II and adequate in stages III, IV and V; in girls, it was adequate in all stages.

**Table 7:** Fat percentage according to pubertal stages (Tanner)

Pubertal stages	Slaughter equation	Gurka equation	BIA	p*	Median difference (Slaughter – BIA)	Median difference (Gurka – BIA)
Stages I-II						
Total population						
(*n* = 78)	9.6 (7.7)	23.8 (7.7)	31.6 (23.4)	< 0.001	-22.0	-7.8
Boys (n = 42)	9.6 (6.6)	21.9 (6.6)	31.9 (28.7)	< 0.001	-22.3	-10.0
Girls (n = 36)	9.8 (8.1)	25.7 (8.1)	29.4 (24.2)	< 0.001	-19.6	-3.7
Stage III						
Total population						
(*n* = 18)	9.0 (6.0)	24.6 (8.5)	20.3 (12.1)	< 0.001	-11.3	4.3
Boys (*n* = 8)	8.5 (3.7)	22.3 (3.7)	18.4 (11.4)	0.001	-9.9	3.9
Girls (*n* = 10)	10.5 (12.1)	29.8 (12.1)	21.1 (20.0)	0.003	-10.6	8.7
Stages IV-V						
Total population						
(*n* = 12)	11.1 (9.0)	19.0 (10.4)	19.6 (17.2)	0.011	-8.5	-0.6
Boys (*n* = 5)	10.6 (12.2)	13.2 (12.2)	20.0 (8.3)	0.002	-9.4	-6.8
Girls (*n* = 7)	11.6 (9.1)	24.3 (9.1)	11.4 (27.3)	0.035	0.2	12.9

Data are presented as median (interquartile range = percentile 75—25). *Kruskal–Wallis; Mann–Whitney U test (stages I and II): Slaughter vs. Gurka *p* < 0.001; Slaughter vs. BIA *p* < 0.001; Gurka vs. BIA *p* = 0.018. Stage III Slaughter vs. Gurka *p* < 0.001; Slaughter vs. BIA *p* = 0.001; Gurka vs. BIA *p* = 0.017. Stages IV and V Slaughter vs. Gurka *p* = 0.001; Slaughter vs. BIA *p* = 0.053; Gurka vs. BIA *p* = 0.713. Comparison of the three groups (total population), Slaughter *p* = 0.735; Gurka *p* = 0.076; BIA *p* = 0.004

Table 8 shows that 70% of the variability of the fat percentage estimated by the Gurka equation is explained by the TSF, 28% by the MUAC and 67% by the arm fat area. Multivariate models were performed with the same variables; those that entered the model were TSF and MUAC, and the coefficient of determination remained similar to that shown in the table.

**Table 8:** Simple regression analysis among anthropometric variables and the fat percentage by the three methods (*n* = 108)

	Slaughter equation			Gurka equation			BIA		
Variables	*r*	*R*^2^	*p*	*r*	*r*^2^	*p*	*r*	*R*^2^	*p*
TSF (mm)	0.943	0.889	< 0.001	0.833	0.694	< 0.001	0.465	0.216	< 0.001
MUAC (cm)	0.669	0.448	< 0.001	0.529	0.280	< 0.001	0.261	0.068	< 0.001
BMI (kg/m^2^)	0.644	0.415	< 0.001	0.527	0.278	< 0.001	0.358	0.128	< 0.001
BMI (Z)	0.585	0.342	< 0.001	0.523	0.274	< 0.001	0.470	0.221	< 0.001
W/A (Z)	0.737	0.543	< 0.001	0.664	0.449	< 0.001	0.367	0.135	0.001
AFA (cm^2^)	0.907	0.826	< 0.001	0.781	0.670	< 0.001	0.414	0.171	< 0.001

*TSF* Triceps skinfold, *MUAC* Mid-upper arm circumference, *BMI* Body mass index, *W/A* Weight for age index, *AFA* Arm fat area

## Discussion

In the present study, it was shown that the estimation of body fat percentage differs among the Slaughter equation, the Gurka equation and BIA, but between Gurka and BIA there are no significant differences. The body composition of children with CP is altered, mainly due to lack of mobility [29]. It has been argued that skinfolds are not accurate in children with CP because when they are reduced in the extremities, they do not necessarily reflect low fat stores since fat in this population tends to accumulate centrally [12, 15]. Despite this, ESPGHAN [30] recommends that fat estimation from skinfolds should be performed routinely as a component of nutritional assessment in children with CP. The fat percentage estimated by BIA and the Gurka equation in our population was very similar to that found by Finbranten et al. (2015) [2] with DXA and the Gurka Eq. (26.3% and 23.8% vs. 28.3% and 30.1%, respectively). The fat percentage estimated by the Slaughter equation in children with CP has been used in several studies [8, 31, 32]. Moreover, as shown in the studies carried out by Finbranten et al. (2015) [2] and Sullivan et al. (2015) [3], this equation underestimates the fat percentage compared to other methods. In our study, the Slaughter equation differed greatly from the other two methods both in the total population and by sex. In a systematic review by Snik et al. (2021) [13], wide limits of agreement (mean difference of -9.6% to -7.1%) were found with the Slaughter equation. It seems that the Gurka equation is more sensitive and accurate for estimating the percentage of fat in boys and girls, since it was the only method that differed between sexes. The estimated fat percentage was higher in the spastic group than in the group with other CP subtypes (ataxic, dyskinetic, hypotonic and mixed). It is important to mention that the Slaughter equation underestimated these findings both in the spastic group and in the group with other CP subtypes with respect to the other two methods. In several studies, the Gurka equation has been shown to adequately estimate the fat percentage in the population with CP [2]. This equation is derived from the Slaughter equation and takes into account a correction factor based on gender, race, pubertal status, and gross motor function [8]. In two studies [2, 28], the fat percentage estimated by this equation was found to have an excellent correlation with that estimated by DXA (r = 0.883 and CCC = 0.86, respectively). However, a study by Rieken et al. (2010) [22] showed that the Gurka equation is not accurate in estimating the fat percentage in children with CP. This might be because, their population consisted only of children with gross motor function levels 4 and 5, and in both our study and that of Finbranten et al. (2015) [2] and Oeffinger (2013) [31], the populations were more heterogeneous. BIA is an appropriate method to estimate the fat percentage in children with CP [30] and has been used in some studies [19, 31, 33]. However, BIA has its own limitations, such as the state of hydration, which is very heterogeneous, varies with age and sex and can give a false result depending on the state of dehydration, which is common in children with CP [13]; in addition, it has a very good correlation with DXA, with differences of less than 2% [31]. The percentage of fat in the most affected group (gross motor function levels 4 and 5) was higher with BIA and the Gurka equation than with the Slaughter equation, and it was higher in this group than in the less affected group (levels 1–3). This finding is very similar to that reported by Finbranten (2015) [2], where an altered body composition was reported according to the level of gross motor function. Likewise, the results are similar to those reported by Whitney et al. (2019) [34], where a higher fat percentage, fat mass and fat mass index was observed in the non-ambulatory population than in the ambulatory population with CP. These findings are also consistent with the notion that stunted children tend to gain fat and become overweight or obese later in life [35]. In our study, this altered body composition was demonstrated through the Gurka equation, which was the only method that showed significant differences between levels 1–3 vs. 4–5. For this reason, the Gurka equation is a more reliable method to estimate the fat percentage in the most affected levels of the CP severity [34]. Slaughter equation correlated positively and moderately with BIA in the spastic group and in the more affected children; it showed a stronger correlation than Gurka equation in the same groups. This finding could be related to the fact that Gurka equation includes an additional correction for the most affected levels (+ 5.1), and 87.9% of our sample belonged to levels 4–5. Besides, Slaughter equation only includes the tricipital and subscapular skinfolds which have a good correlation with the fat percentage. This anthropometric equation should be used carefully to estimate the fat percentage only in the more affected groups because it underestimates the fat percentage even more in the most affected levels [8]. In the study by Rieken et al. (2010) [9], the Gurka equation was shown to be more accurate in estimating the fat percentage in the less affected levels of gross motor function (1–2) and less accurate in the most affected (3–5), perhaps because the size of the population in that study was very small [13]. To our knowledge, this is the first study to analyze body fat percentage based on biological maturity, that included children from all CP subtypes and levels of the GMFCS. It was shown that there was a significant difference in the fat percentage between girls and boys only with the Gurka equation in stages I, II and III. In stages IV and V, there was no significant difference between the sexes, perhaps because the sample size was smaller and could be explained by a type II error. Similar to the results shown in Table 2, we can infer that the Gurka equation is more sensitive than the other two methods for estimating the fat percentage between sexes. It was shown that TSF, AFA, the W/A ratio, MUAC and BMI are the best predictors of fat percentage in children with CP and that they explain most of the variance in fat percentage estimated by the Gurka equation. The variance of the fat percentage estimated by the Gurka equation that was explained by the BMI was exactly the same as that found by Kuperminc et al. (2010) [21] and that regarding the MUAC it was similar; also, the correlation coefficients of the TSF and the AFA were higher than those of the BMI and MUAC. One of the strengths of our study was the sample size, which included subjects with both spastic CP and other CP subtypes. In addition, we included subjects of all levels of gross motor function. One of the limitations was that we did not compare the fat percentage with DXA as gold standard because it was not available, for this reason we estimated the fat percentage by BIA. Another limitation was that the sample size of the stages IV and V of the Tanner classification was small. Another limitation was that the sample size of the group IV-V of the GMFCS was bigger than the group I-III, and finally we had a small sample size of some CP subtypes such as ataxic, dyskinetic, hypotonic and mixed.

## Conclusions

The fat percentage estimated by Gurka equation is similar to that estimated by BIA and it is more appropriate and accurate than Slaughter equation for estimating the fat percentage in children with CP from all subtypes and levels of the GMFCS. The fat percentage can be estimated using the Gurka equation. Longitudinal studies with a larger sample size are required to compare the fat percentage among different age groups.

## Data Availability

Data is available upon request from the corresponding author for the article due to privacy/ethical restrictions.

## Abbreviations

CP: Cerebral palsy
GMFCS: Gross motor function classification system
BIA: Bioelectrical impedance analysis
TSF: Tricipital skinfold
AFA: Arm fat area
W/A: Weight for age
LM: Lean mass
FM: Fat mass
TBW: Total body water
BMD: Bone mineral density
DXA: Dual X-ray absorptiometry
BM: Bone mass
DLW: Doubly labeled water
LL: Leg Lenght
MUAC: Mid upper arm circumference
SSF: Subscapular skinfold
